# Occupational allergy due to seafood delivery: Case report

**DOI:** 10.1186/1745-6673-3-11

**Published:** 2008-05-30

**Authors:** Cornelia S Seitz, Eva B Bröcker, Axel Trautmann

**Affiliations:** 1 Department of Dermatology, Venerology and Allergology, University of Würzburg, Würzburg, Germany

## Abstract

**Background:**

Sensitization to fish or crustaceans requires intensive skin contact and/or airway exposition and therefore especially workers in the seafood processing industry may develop an occupational seafood allergy. However, even in jobs with limited direct exposure, individuals with atopic disposition not using appropriate skin protection are at risk for developing occupational seafood allergy which requires termination of employment.

**Case presentation:**

Due to increasing workload and pressure of time a truck driver in charge of seafood deliveries for 10 years neglected preventive measures such as wearing protective cloths and gloves which resulted in increasing direct skin contact to seafood or mucosal contact to splashing storage ice. Despite his sensitization to fish and crustaceans he tried to remain in his job but with ongoing incidental allergen exposure his symptoms progressed from initial contact urticaria to generalized urticaria, anaphylaxis and finally occupational asthma.

**Conclusion:**

Faulty knowledge and increased work load may impede time-consuming usage of preventive measures for occupational health and safety. In predisposed atopic individuals even minor allergen exposure during seafood distribution may lead to occupational seafood allergy. With ongoing allergen exposure progression to potentially life-threatening allergy symptoms may occur.

## Background

The increase of fish and crustacean allergy in the general population is mainly attributed to the increased consumption of seafood [1]. Independently, workers in the seafood processing industry are a population at increased risk of sensitization due to direct skin contact during handling seafood or inhalation of seafood aerosols e.g. during cooking or when cleaning storage tanks with pressured water [2,3]. Here, we report a truck driver who acquired fish and crustacean allergy by direct skin and mucosal contact due to unprotected handling of fresh seafood. The clinical symptoms of his allergy gradually progressed from contact urticaria to generalized urticaria and later anaphylaxis and occupational asthma.

## Case presentation

A 47-year-old man had started approximately 10 years ago as truck driver delivering fish and other seafood stored on crushed ice. He was in charge of the final quality check of the delivered seafood which included handling of single fish of various species and crustaceans. Plastic skirt and long-sleeved gloves were provided by the employer and were initially invariably used when handling raw seafood. But over the years with increased workload and pressure of time, upon arrival at the client in his rush he frequently did not take the time to change and therefore often times did not wear protecting clothes leading to increased direct contact with fish, crustaceans, the storage ice, and ice water.

Two years prior to presentation at our allergy clinic, the patient first developed itchiness, redness and swelling of the conjunctivae and eyelids one to two minutes after a single drop of ice water splashed into his eye. Although thereafter he tried to strictly avoid skin and mucosal contact, he developed several times within minutes after incidental direct contact to fish, crustaceans, or the storage ice pruritus, erythema and urticaria restricted to the contact sites on the hands and forearms. At one instance with rather intensive contact (carrying a full container by pressing it against the lower abdomen) he developed not only wheals at the contact sites but generalized urticaria. These symptoms subsided within two hours not requiring specific therapy.

One year later when consuming a zander/*Sander *filet which he had been offered by a client, he suffered from generalized urticaria with facial angioedema, nausea, vomiting and defecation. Emergency treatment with intravenously applied fluids, H_1_-antihistamines and corticosteroids lead to complete resolution of anaphylaxis symptoms within two hours. Previously, he had only sporadically consumed seafood, which he had tolerated until then without symptoms. After a second episode of generalized urticaria and angioedema after tasting a small amount of smoked eel/*Anguilla *and a shrimp/*Penaeus *salad he avoided all seafood.

Because of steadily progressive skin symptoms during seafood delivery the truck driver was transferred by the officials of his company to the washing unit of the fish delivery company where amongst others fish transport tanks are cleaned with pressured water. There on his first workday, when cleaning fish transport tanks he developed dyspnoea with exspiratory stridor. Immediately he stopped the cleaning procedure and left the room. Due to the severity of the asthma symptoms emergency treatment with inhalative and subcutaneously applied β-agonists was required.

This case illustrates the progression of food allergy symptoms depending on the site of allergen contact [4]. Presumably, percutaneous sensitization occurred by direct contact of skin and mucosa to fresh seafood stored on crushed ice because these were the initial sites of contact urticaria. With ongoing allergen exposure ingestion of fish and shrimps lead to anaphylaxis before inhalation of seafood-aerosols resulted in asthma symptoms.

On physical examination at our allergy clinic several clinical stigmata supporting atopy were observed: dry skin, keratosis pilaris, pityriasis alba, infrorbital skin fold and white dermographism. Routine laboratory parameters were within normal limits. Total serum immunoglobulin E (IgE) was highly elevated with 1.330 kU/L. Screening prick testing with inhalative allergens such as pollen, cat and dog dander, house dust mites and common food allergens including cow's milk, egg, finned fish, crustacean and hazel nut revealed IgE-mediated sensitizations against herring/*Clupea *(after 20 minutes wheal diameter 15 mm) and shrimp/*Penaeus *(after 20 minutes wheal diameter 22 mm including pseudopods). During the skin testing procedure the patient developed generalized pruritus and dyspnoea. These symptoms subsided after treatment with inhalative β-agonists, intravenously applied H_1_-antihistamines and glucocorticoids. Allergen-specific serum IgE against herring/*Clupea *(f205, CAP System, Pharmacia Diagnostics, Uppsala, Sweden), sardine/*Sardina *(f308), swordfish/*Xiphias *(f312) and shrimp/*Penaeus *(f24) was measured as 29.2, 6.6, 4.2, and 19.2 kU/L, respectively.

The most important heat and ingestion resistant fish allergens are parvalbumines, e.g. the 12 kDa muscle protein Gad c 1 [5]. Approximately 70 % of all patients with fish allergy develop symptoms to several fish species, the remaining 30 % react to only one fish species [6,7]. Tropomyosin (Pen a 1), another muscle protein, is the most important allergen of crustaceans [8]. Cross-reactivities within several crustacean species are common, therefore all crustaceans should be strictly avoided. While cross-reactivity of crustaceans with bony fish (Osteichthyes) is unlikely, cross-reactivity with mollusca (e.g. bivalvia), insecta (e.g. cockroach) and arachnida (e.g. house dust mites) are possible [9].

Due to typical clinical symptoms after exposure to seafood and positive test results diagnosis of IgE-mediated allergy to finned fish and crustaceans was established. The patient's allergy to crustaceans and finned fish was recognized as an occupational disease. Cause for sensitization was probably the ongoing skin contact to native fish and crustaceans for years. Sensitization was facilitated by irritative factors such as wet and cold working conditions (ice water) as well as his atopic background with consecutive disturbance of the physiologic skin barrier function. It has been shown that storage conditions may influence the skin irritancy of fish juice; fish kept on ice for several days enhances frequency and severity of symptoms such as itching, stinging, and erythema [10]. However, the most important risk factor for IgE sensitization against fish and/or crustacean proteins is atopy [11]. In a recent study of employees in the seafood processing industry skin symptoms were predominantly moderate and seldom interfered with working capacity [3]. However, in our case due to the severity of allergy symptoms, including generalized urticaria and asthma, there was a need for termination of the hazardous occupation, which in German jurisdiction is a crucial requirement for approving a condition as an occupational disease [12,13]. In case of accidental allergen contact leading to anaphylaxis the patient received emergency medication including an epinephrine-containing autoinjector and was instructed on the usage [14].

## Conclusion

The significance of the skin for general health is often underestimated. However, in Germany e.g. in 2005 approximately 9.500 cases of occupational skin diseases were among a total of 25.000 approved occupational diseases [13]. Not always the lack of information concerning the necessary protection measures is responsible for the large number of occupational skin diseases. Our patient, a truck driver liked his job as delivery man and initially accurately used protective clothes. However, increased pressure of time and the necessity to constant rush, more and more lead to neglect of necessary protection measures.

## Competing interests

The authors declare that they have no competing interests.

## Authors' contributions

CS carried out testing of the patient and participated in study design and coordination as well as drafting of the manuscript, EB participated in the design of the study and helped to draft the manuscript, AT conceived of the study, participated in its design and coordination and drafted the manuscript. All authors read and approved the final manuscript.

## Consent

Written informed consent was obtained from the patient for publication of this case report. A copy of the written consent is available for review by the Editor-in-Chief of this journal.
